# Visible-light-driven H_2_ production and decomposition of 4-nitrophenol over nickel phosphides

**DOI:** 10.1039/c8ra06770h

**Published:** 2018-10-05

**Authors:** Xing Liu, Lanhua Zhao, Haixia Wang, Hua Lai, Gang Peng, Junhua Li, Zhengji Yi, Kang Chen

**Affiliations:** Key Laboratory of Functional Metal-Organic Compounds of Hunan Province & Key Laboratory of Functional Organometallic Materials of College of Hunan Province, Hengyang Normal University Hengyang 421008 China liuxing1127@sina.com; College of Chemistry and Chemical Engineering, Hunan University Changsha 410082 People's Republic of China; Institue of Pathogenic Biolog, Hunan Provincical Key Laboratory for Special Pathogens Prevention and Control, University of South China Hengyang 421001 People's Republic of China zhaolanhua@126.com

## Abstract

Photocatalytic H_2_ production and photocatalytic decomposition are efficient and economical methods to obtain hydrogen fuel and dispose of organic pollutants. In this paper, amorphous nickel phosphide (Ni_2_P) is synthesized by an extremely simple precipitation method under low temperature. The prepared nickel phosphide was not only used to produce H_2_ in the presence of Eosin Y as sensitizer and triethanolamine (TEOA) as a sacrificial electron donor but also to degrade 4-nitrophenol under visible light illumination. The rate of hydrogen evolution is 34.0 μmol h^−1^ g^−1^ and the degradation efficiency is 25.5% within 4 h at initial 5.0 mg L^−1^ 4-nitrophenol. Probable photocatalytic mechanisms were discussed. The present work is expected to contribute toward the hydrogen evolution and disposal of highly toxic pollutants by cost-effective photocatalytic means.

## Abstract 2

Amorphous nickel phosphide (Ni_2_P) was synthesized by a simple precipitation method. The nickel phosphide was used to produce H_2_ from water with Eosin Y as sensitizer and degrade 4-nitrophenol under visible light illumination.

## Graphical abstract

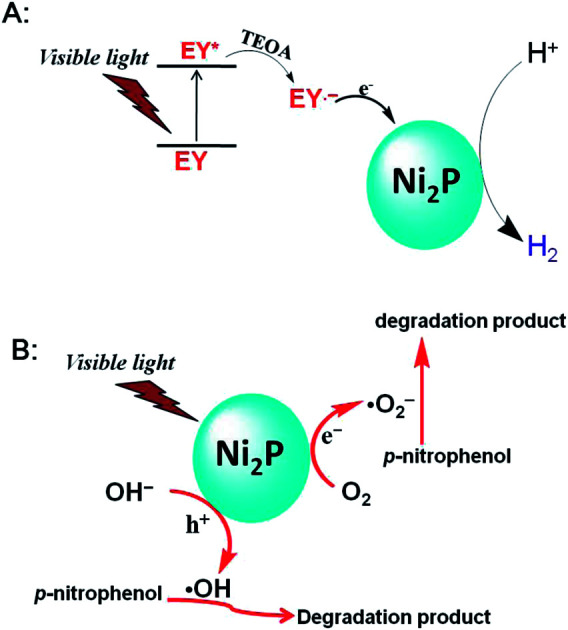

## 1. Introduction

Energy and the environment are two major topics in today's society. Molecular hydrogen (H_2_) is an ideal candidate as a carbon free fuel for the replacement of fossil fuels in the future. The photocatalytic approach is considered as one of the most promising ways to produce H_2_ from water splitting.^1–3^

4-Nitrophenol, used as a raw material or intermediate for the manufacture of agrochemicals, dyes, drugs *etc.*, is one of the most difficult organic contaminants to dispose of, with high toxicity, stability and bioaccumulation.^4–6^ Photocatalytic degradation of organic compounds, including 4-nitrophenol, is one of the most popular approaches of pollution control and disposal.^7–12^

For photocatalytic hydrogen evolution and degradation of organic compounds, many semiconductor photocatalysts, like TiO_2_, can be only activated by UV light due to their high-energy band gaps. The UV light accounts for only *ca.* 4% to 6% of the solar spectrum, while visible light is about 43%. Thus, the development of visible-light-driven photocatalysts is needed.^13–16^

Transition metal phosphides have attracted considerable attention in the fields of electrochemical catalysis due to many advantages such as earth-abundance, high catalytic activity, good stability and facile synthesis.^17–20^ Nickel phosphide is one of the most promising candidates among transition metal phosphides because it is the most active mono-metallic phosphide.^21–23^ Inspired by the high activity of phosphides in electrochemical H_2_ evolution, they were subsequently used as cocatalysts in photocatalytic H_2_ evolution.^24–31^ For example, Cao *et al.* employed Ni_2_P as cocatalysts to modify CdS, and the composite photocatalysts exhibited remarkably improved activity and excellent stability in lactic acid aqueous solution.^32^ However, it is a new attempt for using nickel phosphide to photocatalytic degradation organic contaminants 4-nitrophenol and photosensitized hydrogen evolution from water splitting.

In this work, nickel phosphide (Ni_2_P) material was prepared by a simply precipitation method. The prepared nickel phosphide was used to degrade 4-nitrophenol under visible light illumination and produce H_2_ in the presence of Eosin Y (EY) as sensitizer and triethanolamine (TEOA) as a sacrificial electron donor.

## 2. Experimental

### Synthesis of nickel phosphide

The nickel phosphide was synthesized by a simple precipitation method using nickel acetate (Ni(Ac)_2_·4H_2_O) and sodium hypophosphite (NaH_2_PO_2_) as the starting materials, and all of the used reagents were of analytical grade and were used without further purification. Typically, 5.0 mmol of Ni(Ac)_2_·4H_2_O, 100.0 mmol of NaAc·3H_2_O and a given NaH_2_PO_2_ (the mole ratio of P/Ni = 20/1, 15/1, 10/1 and 5/1) were dissolved into 50.0 mL of distilled water by stirring for 30 min at room temperature, and then the pH of aqueous solution was adjusted to 8.0 with 1.0 M KOH, the reaction system was transferred into a 150 mL three-necked flask and heated at 90 ∼ 95 °C for 12 h. The black products were filtered under vacuum, washed carefully with water and anhydrous ethanol for three times in sequence, and finally dried in a vacuum oven at 60 °C for 5 h. The nickel phosphide catalyst for H_2_ production and 4-nitrophenol degradation was obtained after grinding in an agate mortar.

### Photocatalytic test

Photocatalytic reaction was conducted at room temperature, and a high pressure Hg lamp (150 W) was used as the light source, which was equipped with a cutoff filter (*λ* > 420 nm) to remove the radiation below 420 nm. The IR fraction of the beam was removed by a cool water filter to ensure illumination of visible light only.

In a typical H_2_ production experiment,^33^ 0.10 g of nickel phosphide catalyst was added into 80.0 mL of aqueous solution containing TEOA (9.5 × 10^−2^ mol L^−1^) and EY (pH was adjusted as 7.0 with hydrochloric acid). Before irradiation, the catalyst was dispersed in an ultrasonic bath for 5 min and N_2_ was bubbled through the reaction mixture for 30 min to remove oxygen. The top of the cell was sealed with a silicone rubber septum. Sampling was operated intermittently through the septum during experiments. The amount of hydrogen evolved was determined on a gas chromatograph (TCD, 13X molecular sieve column, N_2_ gas carrier).

In a 4-nitrophenol degradation experiment, 0.10 g of nickel phosphide photocatalysts was suspended into 100 mL of 4-nitrophenol aqueous solution and stirred under dark for 30 min to reach adsorption equilibrium. The mixed solution was irradiated under continuous stirring. Samples were taken out every 60 min, and centrifuged at speed of 10 000 rpm for 10 min in order to remove the photocatalysts, the obtained clear solution was analyzed using spectrophotometric method on a 7SS2 spectrophotometer (made in Shanghai, China), and the absorbance was measured at the wavelength of 315 nm.^34^

### Characterization and instruments

X-ray diffraction (XRD) analysis was carried out using a BRUCKER D8 polycrystalline X-ray diffractometer with Cu Kα radiation as the X-ray source (*λ* = 0.15406 nm) at a scan rate of 6° min^−1^. Scanning electron microscopy (SEM) image was taken on a EVO-10 (Carl Zeiss AG, Germany) working at 30 kV, the sample was mounted onto carbon adhesive pad attached to aluminum stub. X-ray photoelectron spectroscopy (XPS) analyses were performed using an ESCALAB250xi photoelectron spectrometer (Thermon Scientific) with a monochromatized Mg Kα X-Ray resource (150 W). The C 1s peak (284.8 eV), was used as an internal reference for absolute binding energy.

## 3. Results and discussion

**Fig. 1:**
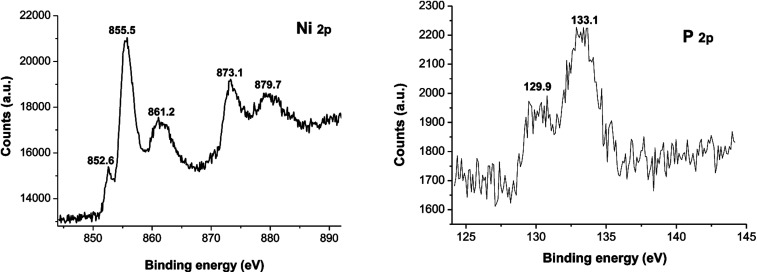
X-ray photoelectron spectroscopy (XPS) spectra of Ni_2_P.

XPS spectra of Ni_2_P were shown in Fig. 1, from the element measurement from XPS, the atom ratio of Ni/P is 2, this confirms that the prepared nickel phosphide is Ni_2_P. For Ni 2p region, three peaks at 852.6, 855.5 and 861.2 eV, which is ascribed to Ni^*δ*+^ (0 < *δ* < 2) in Ni_2_P, oxidized Ni species (Ni^2+^) and the satellite of the Ni 2p_1/2_ peak, respectively.^29^ And the peaks at 873.1 and 879.7 eV is corresponding to Ni^*δ*+^ in Ni_2_P, oxidized Ni species and the satellite of the Ni 2p_3/2_ peak, respectively. For the P 2p, the peak at 129.9 eV is a mark of metal–P bonds in metal phosphides (*i.e.* Ni_2_P),^29^ while the peak at 133.1 eV can be attributed to the oxidized P species due to air contact.

**Fig. 2:**
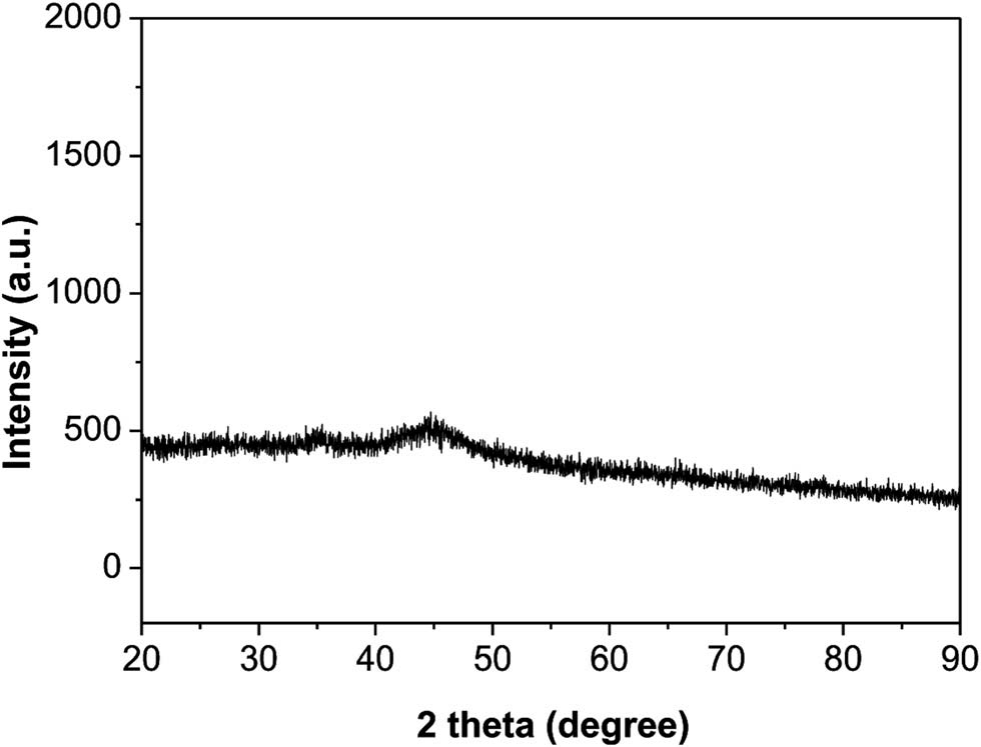
XRD pattern of Ni_2_P.

**Fig. 3:**
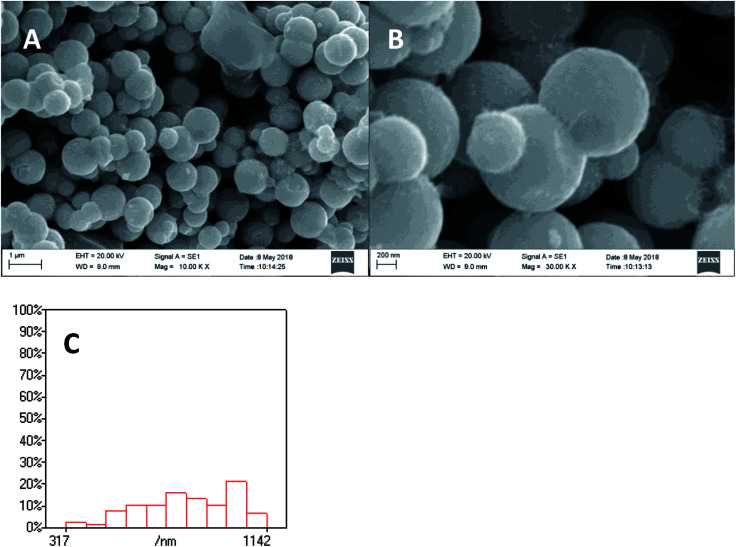
SEM image (A and B) and particle size distribution (C) of Ni_2_P.

As shown in Fig. 2, the XRD diffraction pattern of Ni_2_P showed no obvious diffraction peaks, which indicates that the Ni_2_P has an amorphous structure.^30^ The morphology of Ni_2_P is revealed using SEM. As shown in Fig. 3A and B, the Ni_2_P sample is composed of nanospheres with various sizes, and nanospheres are stacked together. The statistical particle size distributions of Ni_2_P were shown in Fig. 3C, the average diameter of Ni_2_P nanospheres is 800 ± 30 nm.

**Fig. 4:**
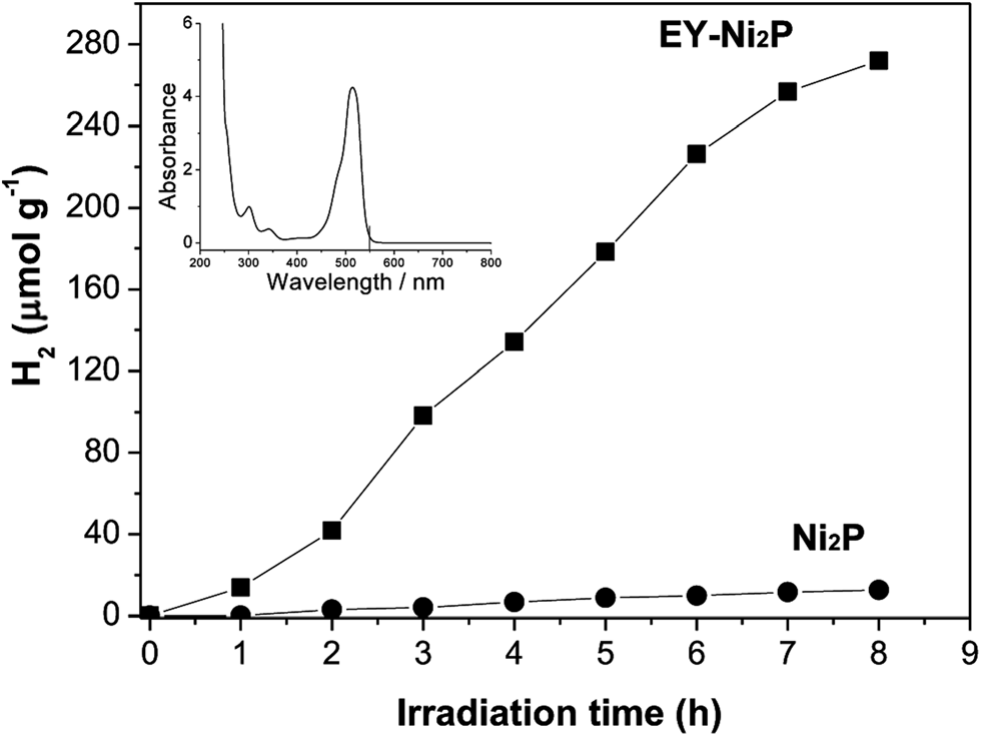
Time courses of photocatalytic H_2_ production of EY–Ni_2_P and Ni_2_P. Conditions: 0.10 g Ni_2_P, 9.5 × 10^−2^ mol L^−1^ TEOA, light source: 150 W high pressure Hg lamp, *λ* > 420 nm. The inset is UV-Vis absorption spectra of EY.

A noble-metal-free hydrogen production system was constructed with the as-prepared Ni_2_P as a catalyst, EY as a photosensitizer, and TEOA as a sacrificial electron donor. It is observed from Fig. 4 that Ni_2_P (without photosensitizer) displays very low photocatalytic H_2_ evolution activity, while no H_2_ can be observed in the system containing only EY (without nickel phosphides). And the EY–Ni_2_P (EY sensitized Ni_2_P) system has a remarkable activity under the same experimental condition, the rate of hydrogen generation is 34.0 μmol h^−1^ g^−1^. The result indicates that EY can markedly enhance the hydrogen generation activity.

**Fig. 5:**
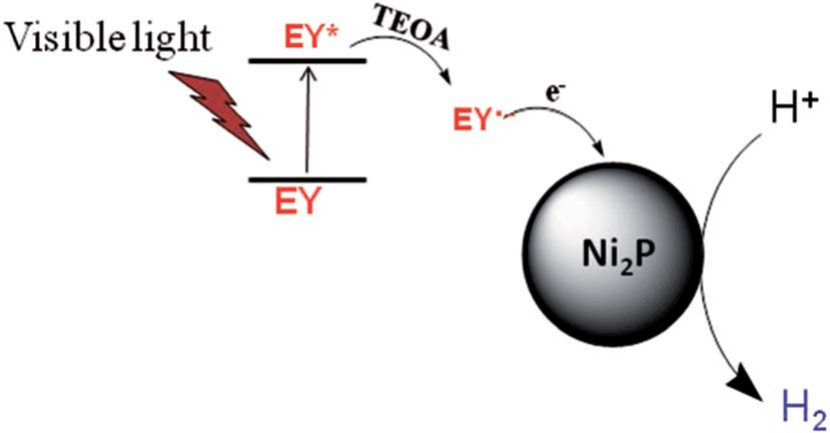
Schematic illustration of probable mechanism of photosensitized hydrogen production. Noted that EY*, EY˙^−^, TEOA represent the triplet excited state of Eosin Y, the radical anion of Eosin Y and triethanolamine, respectively.

From the UV-Vis absorption spectra of EY (the inset of Fig. 4), EY can strongly absorb visible light during the range of 450 ∼ 560 nm with the absorption centre at 515 nm. When the EY photosensitizer is irradiated by visible light, EY absorbs photons, and electrons in the HOMO (highest occupied molecular orbital) can be excited to the LUMO (lowest unoccupied molecular orbital), and resulting in excited EY*. EY* is prevailingly quenched reductively by electron donor TEOA to form radical EY˙^−^ anion, the surplus electrons of EY˙^−^ can be trapped by Ni_2_P, and then the electrons react with H^+^ adsorbed on Ni_2_P to generate H_2_ gas. A possible reaction mechanism of the hydrogen evolution is shown in Fig. 5. However, the produced EY˙^−^ anion could also undergo self-degradation by debromination, the self-degradation decreases the sensitization activity.^33,35^ In Fig. 4, it can be found that the H_2_ evolution activity decreased in the last two hours, this phenomenon suggests the above discussion.

**Fig. 6:**
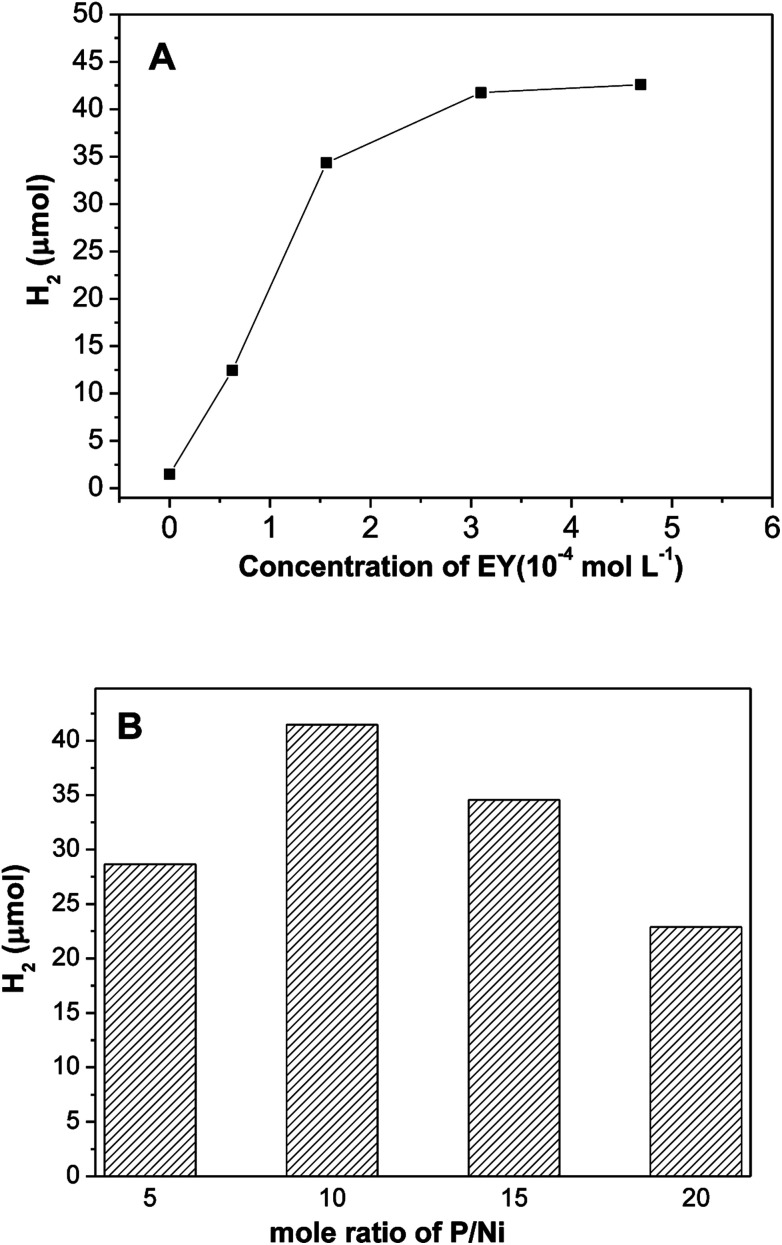
Effect of (A) initial EY concentration and (B) mole ratio of initial P/Ni on hydrogen evolution of EY sensitized Ni_2_P.

Based on the above discussions, the EY plays a critical role in number of photo-induced electrons, which results in the photocatalytic hydrogen evolution. Fig. 6A shows the impact of EY concentration on hydrogen evolution. The photocatalytic activities increases first and then declines with increase of EY concentration, and reached a maximum at 3.1 × 10^−4^ mol L^−1^. If no dye is added, only a little evolved hydrogen is observed, this demonstrates that hydrogen generation is indeed driven by dye sensitization. When concentration of EY increases from 0 to 3.1 × 10^−4^ mol L^−1^, the antenna effect of dye to absorb light is boosted, more and more radical EY˙^−^ anion generates, thus the hydrogen evolution is improved. Nevertheless, when the concentration of EY is extremely high, numerous free dye molecules in solution absorb the input light but would not contribute to hydrogen evolution reaction. Moreover, greater collisional deactivation of the ^3^EY* excited state happens due to serried dye molecules,^35,36^ so the activity of hydrogen evolution would not increase. Fig. 6B reveals the effect of mole ratio of initial P/Ni on hydrogen evolution over EY sensitized Ni_2_P, the highest photo-activity occurs at P/Ni = 10. All Ni_2_P samples prepared with different ratio of initial P/Ni has exactly similar XRD diffraction patterns, indicating that they are amorphous. When the ratios of initial P/Ni are 5/1, 10/1, 15/1, 20/1, the BET specific surface areas of the prepared nickel phosphides samples are 18.9, 27.7, 23.1, 20.5 m^2^ g^−1^, respectively. This suggests that the specific surface areas of nickel phosphide with ratio of initial P/Ni = 10 is largest. Since the hydrogen evolution reaction is surface-dependent, a large surface area should provide more surface active sites for the adsorption of reactants, making the photocatalytic H_2_ production more efficient.

**Fig. 7:**
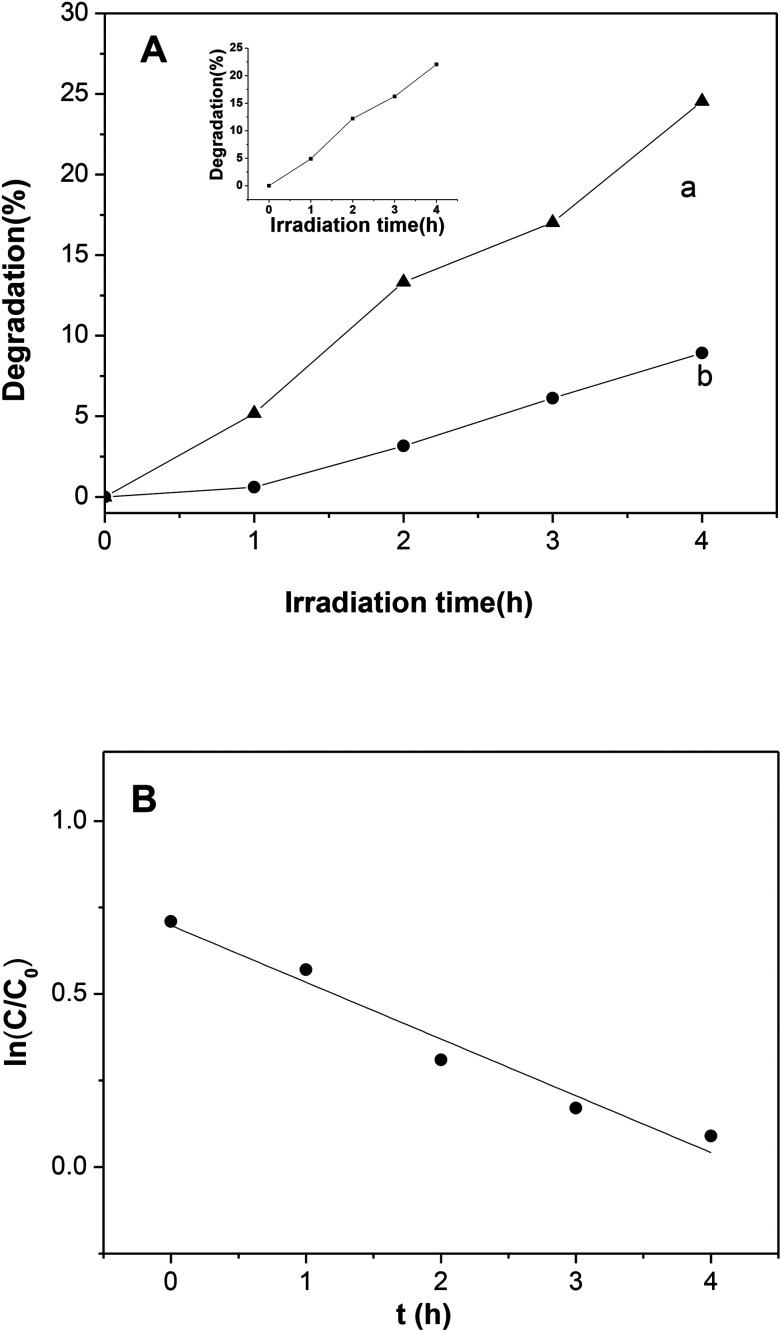
(A) Degradation curves of *p*-nitrophenol over (a) Ni_2_P, (b) self-degradation without photocatalysts. The inset is second cycle for Ni_2_P. (B) Plots of pseudo-first-order kinetics for photocatalytic degradation over Ni_2_P. Experimental conditions: Ni_2_P was prepared at mole ratio of P/Ni = 10/1, initial concentration of 4-nitrophenol is 5.0 mg L^−1^.

The photocatalytic performance of Ni_2_P was also evaluated by degradation of 4-nitrophenol under visible light illumination. As shown in Fig. 7A, self-degradation of 4-nitrophenol is almost negligible when the initial concentrations of 4-nitrophenol is 5 mg L^−1^. In the presence of the Ni_2_P, the remarkable degradation was found, and the degradation efficiency is 25.5% within 4 h. In 4-nitrophenol degradation, the activity decrease in second cycle degradation is not obvious (see the inset of Fig. 7A), this indicates that nickel phosphides is stable. A control experiment reveals that the degradation of 4-nitrophenol over Ni_2_P under dark condition is also negligible, this result confirms that the degradation is indeed a photochemical reaction. The degradation efficiency of 4-nitrophenol over Ni_2_P is not high, which can be explained as following: firstly, charge recombination happens easily in Ni_2_P when it is excited by visible light, thus the generated oxidative species (see the following discussion) is limited. Secondly, the light source in our experiments was a 150 W high pressure Hg lamp (equipped with a cutoff filter (*λ* > 420 nm)), compared with xenon lamp, its light intensity was very low, so the photocatalytic activity is low. In addition, the BET specific surface areas of prepared nickel phosphides is relatively small (<28 m^2^ g^−1^), so photocatalytic active sites are limited.

According to previous study, the kinetic behavior of the photocatalytic reaction obeys pseudo-first-order kinetics. To confirm this speculation, the natural logarithm of 4-nitrophenol concentration ln(*C*/*C*_0_) is plotted as a function of irradiation time (*C* and *C*_0_ are the concentrations of 4-nitrophenol at time *t* and zero, respectively). The kinetics for the photodegradation of 4-nitrophenol was shown in Fig. 7B, the regression curve is roughly linear, indicating that the kinetics of 4-nitrophenol degradation over the photocatalyst is indeed pseudo-first-order, and the reaction rate constant *k* was calculated to be 0.16 h^−1^.

As is known, the photocatalyst Ni_2_P can be directly excited to generate electron (e^−^)–hole (h^+^) pairs after visible light illumination. The photoinduced holes react with surface-bound H_2_O and produce the hydroxyl radical ·OH species that are extremely strong oxidant for the mineralization of organic pollutions, including 4-nitrophenol. Meanwhile, the photoinduced electrons can react with the adsorbed molecular oxygen to yield superoxide radical ·O_2_^−^, which combine with H^+^ to produce HO_2_˙. The HO_2_˙ can further react with the trapped electrons to generate ·OH radicals. To further study the photocatalytic mechanism and identify the main oxidative species (h^+^, ·OH and ·O_2_^−^) in the photocatalytic process, we studied the effect of active-species scavengers on the degradation of 4-nitrophenol.^34^ It is found that ·OH and ·O_2_^−^ radicals are the main reactive species in the photocatalytic degradation. The intermediate products of 4-nitrophenol photodegradation include 4-nitrocatechol, 1,2,4-benzenetriol, hydroquinone, which have been demonstrated by previous works containing ours.^34,37,38^ The ultimate products are carbon dioxide, water, nitrate after complete mineralization.

**Fig. 8:**
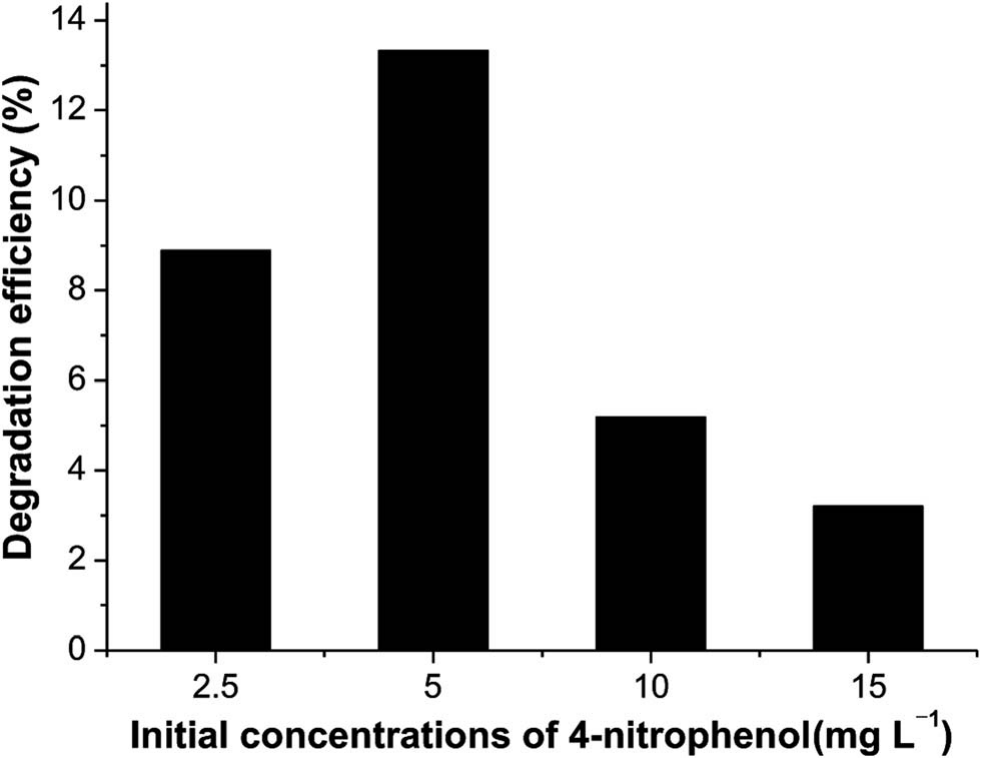
Photocatalytic degradation of 4-nitrophenol over Ni_2_P at different initial concentration. Experimental conditions as in Fig. 7.

Fig. 8 illustrates the performance of Ni_2_P when the initial concentrations of 4-nitrophenol vary from 2.5 to 15.0 mg L^−1^. The dependence of the photodegradation yield of 4-nitrophenol on the initial concentration may be based on the fact that the degradation reaction mainly occurs on Ni_2_P surface. On the surface of catalyst, the reaction happens between ·OH radicals and 4-nitrophenol molecules. When the initial 4-nitrophenol concentration is low, the transfer rate plays an important role, the adsorption of 4-nitrophenol on the catalyst surface increases as the initial concentration increases, which in favour of the subsequent photocatalytic degradation. However, with further increase of 4-nitrophenol concentration, the rate of photocatalytic degradation decreases due to the reduced ratio of ·OH (or ·O_2_)/4-nitrophenol. Therefore, the highest photocatalytic degradation efficiency is obtained when the initial 4-nitrophenol concentration is 5.0 mg L^−1^. This finding demonstrates that the degradation kinetics of 4-nitrophenol is indeed not of simple first order but pseudo first order within the experimental range, which is consistent with many previous reports.^37–40^

## 4. Conclusions

In conclusion, nickel phosphide was synthesized by a simple precipitation method, XRD and SEM characterization results demostrate that the Ni_2_P is an amorphous nanospheres. Eosin Y sensitized nickel phosphide displays relatively high photocatalytic activity for hydrogen evolution with the rate of 34.0 μmol h^−1^ g^−1^. The Ni_2_P also exhibits 4-nitrophenol degradation under visible light illumination with degradation efficiency of 25.5% within 4 at initial 5.0 mg L^−1^ 4-nitrophenol concentration. Probable mechanisms of photocatalytic hydrogen evolution and photo-degradation of 4-nitrophenol were discussed.

## Conflicts of interest

There are no conflicts to declare.

## Supplementary Material
